# Relative synonymous codon usage and codon pair analysis of depression associated genes

**DOI:** 10.1038/s41598-024-51909-8

**Published:** 2024-02-12

**Authors:** Rekha Khandia, Pankaj Gurjar, Mohammad Amjad Kamal, Nigel H. Greig

**Affiliations:** 1 Department of Biochemistry and Genetics, Barkatullah University, Bhopal, 462026 MP India; 2 Centre for Global Health Research, Saveetha Medical College and Hospital, Saveetha Institute of Medical and Technical Sciences, Saveetha University, Chennai, Tamilnadu India; 3 Department of Science and Engineering, Novel Global Community Educational Foundation, Hebersham, NSW, Australia; 4 Joint Laboratory of Artificial Intelligence in Healthcare, Institutes for Systems Genetics and West China School of Nursing, Frontiers Science Center for Disease-related Molecular Network, West China Hospital, Sichuan University, Chengdu, China; 5 King Fahd Medical Research Center, King Abdulaziz University, Jeddah, 21589 Saudi Arabia; 6 Department of Pharmacy, Faculty of Allied Health Sciences, Daffodil International University, Dhaka, 1207 Bangladesh; 7 Enzymoics, Novel Global Community Educational Foundation, 7 Peterlee place, Hebersham, NSW, 2770 Australia; 8 Translational Gerontology Branch, Intramural Research Program, National Institute on Aging, NIH, Baltimore, MD 21224 USA

**Keywords:** Genetic interaction, Medical genetics

## Abstract

Depression negatively impacts mood, behavior, and mental and physical health. It is the third leading cause of suicides worldwide and leads to decreased quality of life. We examined 18 genes available at the genetic testing registry (GTR) from the National Center for Biotechnological Information to investigate molecular patterns present in depression-associated genes. Different genotypes and differential expression of the genes are responsible for ensuing depression. The present study, investigated codon pattern analysis, which might play imperative roles in modulating gene expression of depression-associated genes. Of the 18 genes, seven and two genes tended to up- and down-regulate, respectively, and, for the remaining genes, different genotypes, an outcome of SNPs were responsible alone or in combination with differential expression for different conditions associated with depression. Codon context analysis revealed the abundance of identical GTG-GTG and CTG-CTG pairs, and the rarity of methionine-initiated codon pairs. Information based on codon usage, preferred codons, rare, and codon context might be used in constructing a deliverable synthetic construct to correct the gene expression level of the human body, which is altered in the depressive state. Other molecular signatures also revealed the role of evolutionary forces in shaping codon usage.

## Introduction

Depression is acknowledged as a worldwide major public health concern by numerous international agencies and national governments^1^. According to the World Health Organization in 2016, depression accounts for 10% of the non-fatal disease burden worldwide^2^. It has an hereditary element, and can result from genetic and environmental influences. Depression represents a complex polygenic and multifactorial disorder where many genetic variants, each with a small or unnoticeable impact, combine to contribute to the resulting phenotype^3^. Genome-wide association studies (GWAS) have identified 178 genetic risk loci and 223 independently significant SNPs^4^. There are almost 1500 symptom combinations that fulfil the diagnostic criterion for depression, and any two patients of depressive disorder may, very likely, not have common symptoms^5^. Gender also has an impact, and women are nearly twice as likely as men to be diagnosed with depression. In this light, a greater genetic understanding of depression is needed to help achieve improvements in diagnosis and treatment^6^.

Convergent preclinical and clinical research data have revealed significant correlations among stress, depression, and epigenetic abnormalities. Depressive disorders are widespread, disabling, and costly illnesses that are linked to a decreased role in functioning and quality of life and an increase in medical comorbidity and mortality^7^. Numerous studies on depression have focused on mutations and the genetic composition of genes. In contrast, there has been minimal analysis of the codon usage bias (CUB) of genes associated with depression. CUB is the unequal use of synonymous codons of an amino acid in which some codons are utilized more often than others. Hence CUB analysis can prove valuable in aiding our understanding of molecular biology, genetics, and functional regulation of gene expression. Computational evaluation on codon bias has been of recent research interest to determine the role of codon preference in disorders with a genetic component, such as in anxiety, Alzheimer's disease, and others.

There are 61 codons that encode for amino acids and, excluding methionine and tryptophan, two or more codons encode each single amino acid, and such codons are called synonymous codons. Codons encode a total of 20 amino acids, and it is now well-established that synonymous codon usage is not random^8^. Although the amino acid sequence is not altered, changes are evident in mRNA secondary structure, and its stability^9^. With that, usage of cognitive tRNA is also affected. As a result, these alterations, previously thought to be phenotypically silent and frequently overlooked in investigations of human genetic diversity, are gaining the scientific community's attention as a reason behind several medical disorders. These synonymous codon changes may significantly alter gene expression levels^10^. Stop codon readthrough (SCR), for example, is a known phenomenon where translation is continued beyond the stop codon, and protein isoforms are generated. The SCR is found to be associated with the codon context, and UGA is the leakiest stop codon^11^. In the context of physiological consequence, for the water channel Aquaporin 4 (AQP4), agents that stimulate an unusual SCR event were found to mediate improved Aβ clearance and, thereby, provide insight as well as a new potential therapeutic strategy for Alzheimer’s disease^12^. Rare codons can cause ribosomes to pause on a mRNA during translation and mediate premature chain termination. Indeed, some genetic conditions, like cystic fibrosis, may arise from incorrect stop codons in genes^13^. Bias in codon usage impacts mRNA stability and translation fidelity^14^. In the light of these facts, we hypothesize role of CUB in depression may, in part, underpin disease expression. A greater understanding of these patterns may aid define new potential targets and/or markers for human disorders^9,10^, such as depression.

In this regard, whereas various studies have appraised point mutations and variant analysis of genes involved in depression; to our knowledge, no study has yet been conducted on codon pattern analysis of such genes. Therefore, in the present study, our primary goal was to evaluate the codon preference for expression-associated genes. Additionally, skew, neutrality, parity, protein properties, gene expression, codon pair, and codon context analyses were also assessed. Our overall analysis aids in revealing different molecular patterns in the depression-associated genes to help expose their molecular signatures.

## Results

### Result of pathway analysis

Pathway analysis for the envisaged genes was conducted through the PANTHER knowledgebase to understand the involvement of genes in various vital pathways. A total of 12 pathways were assigned to the 18 genes, which were associated with 5-Hydroxytryptamine biosynthesis, 5HT1 type receptor-mediated signaling pathway, 5HT2 type receptor-mediated signaling pathway, 5HT3 type receptor-mediated signaling pathway, 5HT4 type receptor-mediated signaling pathway, Adrenaline and noradrenaline biosynthesis, Bupropion degradation, Dopamine receptor-mediated signaling pathway, Heterotrimeric G-protein signaling pathway-Gi alpha and Gs alpha mediated pathway, Huntington disease, Metabotropic glutamate receptor group II pathway and Nicotine degradation. Pathways analysis shows that these genes are mainly associated with signal transduction and metabolic processes.

### Compositional analysis

Depression-related testing for genes was searched from the Genetic Testing Registry (GTR), National Center for Biotechnology Information Search database. The tests gtr/tests/508,961 by Assurex Health Inc, gtr/tests/569,407 by genomind Professional PGx Express CORE Anxiety & Depression, and gtr/tests/579,485 by Intergen Genetic Diagnosis and Research Centre presented a panel of 18 genes that are evaluated for the presence of depressive disorders. Different gene genotypes are available based on the SNPs; however, we accessed only the ‘reference’ coding gene sequences from the NCBI nucleotide database. Although a larger number of genes is preferable to support statistical analyses, this was the available total number of genes in the accessible panel targeted to a depression diagnosis and, hence, 18 gene sequences were obtained (for specifics, see Table 1).

**Table 1:** Depression associated genes evaluated for codon pattern analysis: their regular functions and roles during depression along with their modulated expression and SNP data.

S. no	Gene name	Regular functions	Ref	Observed modulation in expression	Expression modulation Ref	SNP data related to depression	Outcome of SNP	SNP related to allelic changes	References
1	Brain-derived neurotrophic factor (BDNF)	Trophic factor underpinning neuron survival—with decreased expression in depressive disorder patients	^15^	Under expression	^15^	rs6265	Drug response towards escitalopram	C > T	^16^
2	Catechol-O-Methyltransferase (COMT) gene	Key catecholamine inactivating enzyme—degrading neurotransmitters like dopamine, norepinephrine and epinephrine	^17^	Under expression	^18^	rs4680	Drug response towards escitalopram	G > A	^16^
3	Cytochrome P450 1A2 (CYP1A2)	Involved in metabolism of estrogens and many exogenous compounds, including caffeine	^19^	–	–	rs4646425, rs4646427 , rs762551	Treatment efficacy and side effects of paroxetine	C > T, T > C, C > A,G	^20^
4	Cytochrome P450 2B6 (CYP2B6)	Metabolism of anticancer, antidepressant, antimalarial, anti retrovirals	^21^	–	–	rs3745274	Genotype GG associated with increased risk of Depression	G > A,C,T	^22^
5	Cytochrome P450 family 2 subfamily C member 19 (CYP2C19)	Implicated in depression severity and treatment response	^23^	Over expression is associated with depressive symptoms and hippocampal homeostasis impairment	^24^	–	–	–	–
6	Cytochrome P450 family 2 subfamily C member 9 (CYP2C9)	Its genetic polymorphism may be related to a major depressive disorder	^25^	Under expression	^26^	–	–	–	–
7	Cytochrome P450 family 2 subfamily D member 6 (CYP2D6)	Metabolism of antidepressants, including selective serotonin reuptake inhibitors	^27^	–	–	rs3892097	Higher dose of antidepressant required for genotype CC Genotypes	C > A,G,T	^28^
8	Cytochrome P450 family 3 subfamily A member 4 (CYP3A4)	Metabolism of dietary compounds, prescribed drugs and xenobiotics, steroid hormones, and bile acids	^29^	Under expression	^26^	rs2740574, rs4646437	Genotype T associate with associated with clearance of risperidone, Allele G associate with associated with clearance of risperidone	C > A,G,T, G > A	^30^
9	Cytochrome P450 family 3 subfamily A member 5 (CYP3A5)	Metabolism of steroid hormones and vitamins	^31^	–	–	rs776746	Genotype T associated with clearance of risperidone in people with Bipolar Disorder, Depression	T > C	^30^
10	Major histocompatibility complex, class I, A (HLA-A)	MHC antigen specific to humans	^32^	–	–	–	–	–	–
11	Major histocompatibility complex, class I, B (HLA-B)	MHC antigen specific to humans	^32^	–	–	–	–	–	–
12	Serotonin receptor 2A (HTR2A) gene	Associated with withdrawn behaviour and with hypertension risk	^33^	Under expression	^34^	rs7997012	Genotype AA is associated with increased likelihood of response when treated with citalopram, Allele A is associated with increased likelihood of response when treated with citalopram	A > C,G,T	^35,36^
13	Melanocortin 4 receptor (MC4R)	Regulator of energy homeostasis	^37^	–	–	rs17782313	Association with depressed mood	T > A,C	^38^
14	Methylenetetrahydrofolatereductase (MTHFR)	Folate and homocysteine metabolism	^39^	Under expression leads to mood and anxiety disorders	^40^	rs1801133	Genotype AG is associated with increased response to l-methylfolate	G > A,C	^41^
15	Solute carrier family 6 member 4 (SLC6A4)	Encodes for serotonin transporter (5-HTT)	^42^	Increased methylation and under expression leads to lifelong depression	^43^	rs140700, rs6354, rs25528, rs25531	Association between depression and CpG methylation	C > A,G,T, G > A,C,T, G > A,T, T > C,G	^44^
16	UDP-glucuronosyltransferase 2B15 (UGT2B15)	Glucuronidation	^45^	–	–	rs1902023	Genotype AA is associated with decreased oxazepam oral clearance	A > C	^46^
17	UDP glucuronosyl-transferase family 1 member A1 (UGT1A1)	Glucuronidation of bilirubin	^47^	Overexpression during stress	^48^	–	–	–	–
18	Tryptophan hydroxylase 2 (TPH2)	Isozyme of tryptophan hydroxylase, present in the serotonergic neurons of the brain	^49^	–	–	rs1487278, rs1843809	Allele C is associated with increased response to mirtazapine and venlafaxine, Genotype TT is associated with decreased likelihood of aggression and Depression	T > C, G > A,T	^50,51^

Our compositional analysis of genes involved in depression revealed that GC3 content, which is an indicator of codon bias^52^, was highest amongst all other compositional parameters. Average %A, %C, %T and %G composition was 24.39%, 26.17%, 23.66% and 25.75%, respectively. In occurrence, these nucleotides appear in the order of %C > %G > %A > %T. At codon position one nucleotide composition %T1 (18.67%), at codon position two %G2 (17.82%) and at the third codon position %A3 (16.42%) were least, and %GC3 content varied between 41.80% and 83.82%.

### GC content (GC12 and GC3) effects on gene length

**Figure 1:**
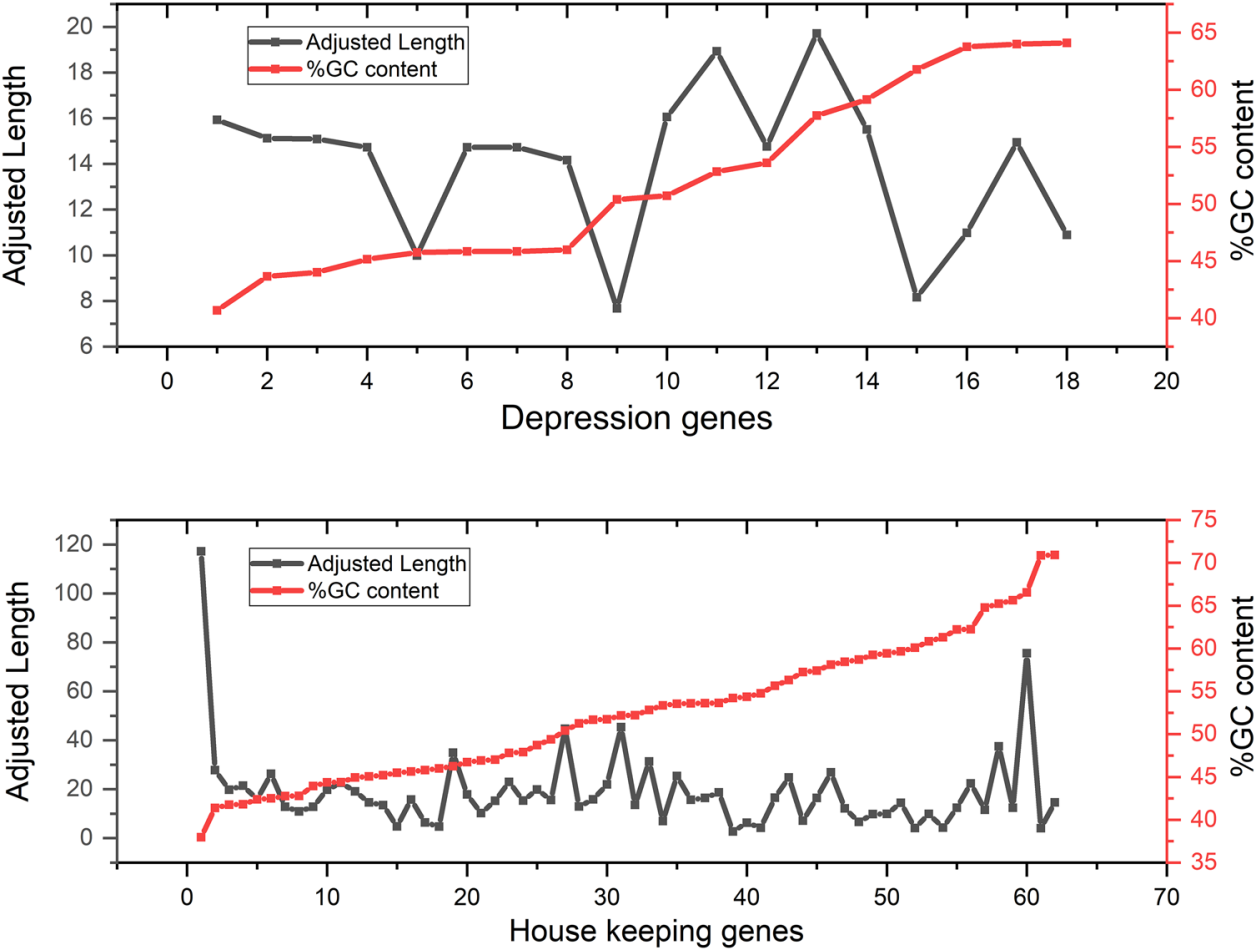
Length vs %GC3 content in depression (top) and housekeeping (bottom) genes.

The coding-sequence lengths possess an evolutionary meaning in relation to GC content compositional variations in DNA. An analysis of the genome database revealed a richness of GC in the longest coding sequences in vertebrates and prokaryotes, with the additional observation that the shorter versions of these are GC poor^53^. A Pearson correlation coefficient (r) was obtained based on the linear correlation between the two data sets. This analysis revealed a lack of correlation between length and GC components %GC12 and %GC3, which indicated no dependency of %GC content on lengths of genes. A trend was observed that among all 18 evaluated genes, most of the genes had a size between 1350 and 1650 bp. Furthermore, in all the genes, %GC3 content was higher than %GC12. Gene lengths were normalized by dividing them by 100 to be comparable with the percent GC composition. A depiction of normalized gene length and %GC3 content is given in Fig. 1. To evaluate correlation trends between length and %GC content, we additionally appraised the correlation between the adjusted length and %GC content of a set of 62 housekeeping genes. We found that length negatively correlates with %GC3 (Pearson correlation coefficient r = -0.263, p < 0.05) in housekeeping genes (Supplementary Table S1).

### Dinucleotide ratio analysis

Dinucleotides CpG, GpT, and TpA were either underrepresented or randomly presented (odds ratio < 1.6) in all the genes envisaged. On the other hand, ApG, CpT, GpA, and TpG dinucleotides were either overrepresented or randomly presented (odds ratio > 1.6).

### RSCU analysis shows preference of GC ending codons

**Figure 2:**
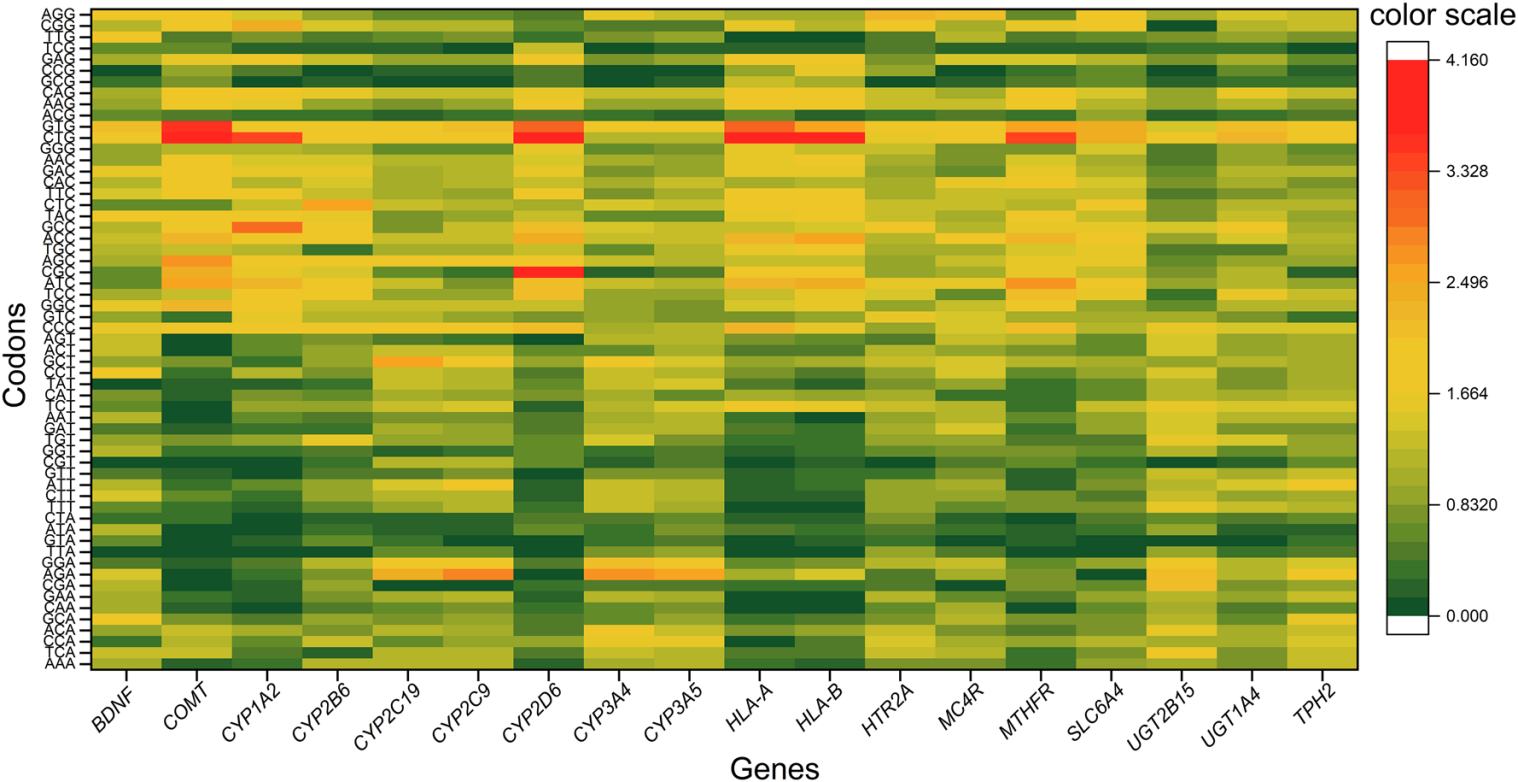
RSCU values of different codons in 18 depression associated gene sets shows an underrepresentation of A/T ending codons.

The overall RSCU analysis revealed that GC ending codons were preferred over AT ending codons. CTG and GTG codons were the most overrepresented codons, whereas TTA, GTA, ATA, CTA, CGT, ACG, GCG, CCG, and TCG codons were the most underrepresented codons (Fig. 2). RSCU values of depression associated genes are shown in Table 2. To determine the correlation trends between length and %GC content, we further sought a correlation between adjusted length and %GC content of a set of 62 housekeeping genes. Also, we compared RSCU values of depression-associated genes with the RSCU values of housekeeping genes, and, based on t-test, it was evident that codon usage was significantly different (t = 3.58, p < 0.0001) for codon GTA. In addition to this, codons GTG, CCC, GAT, and GAC also differed at a 10% significance level (Table 3).

**Table 2:** RSCU values of individual genes.

GENE	BDNF	COMT	CYP1A2	CYP2B6	CYP2C19	CYP2C9	CYP2D6	CYP3A4	CYP3A5	HLA-A	HLA-B	HTR2A	MC4R	MTHFR	SLC6A4	UGT2B15	UGT1A4	TPH2
TTT	0.57	0.33	0.24	0.62	0.97	1.13	0.30	1.25	1.00	0.00	0.00	0.96	0.67	0.71	0.70	1.58	1.25	1.14
TTC	1.43	1.67	1.76	1.39	1.03	0.88	1.70	0.75	1.00	2.00	2.00	1.04	1.33	1.29	1.30	0.42	0.75	0.86
TTA	0.00	0.00	0.00	0.10	0.64	0.62	0.00	0.81	0.84	0.00	0.00	0.95	0.45	0.09	0.10	0.90	0.38	0.51
TTG	2.00	0.43	0.79	0.48	0.64	0.72	0.36	0.71	0.94	0.00	0.00	0.53	1.20	0.46	0.69	0.80	0.86	0.77
CTT	1.43	0.57	0.39	0.86	1.18	1.14	0.18	1.32	1.13	0.21	0.21	0.84	0.90	0.74	0.49	1.30	0.86	1.02
CTC	0.57	0.57	1.28	2.57	1.29	1.24	1.09	1.42	1.31	1.93	1.71	1.37	1.35	1.20	1.87	0.70	1.14	1.15
CTA	0.29	0.29	0.10	0.19	0.21	0.21	0.46	0.51	0.66	0.21	0.21	0.74	0.15	0.09	0.49	0.60	0.48	0.64
CTG	1.71	4.14	3.44	1.81	2.04	2.07	3.91	1.22	1.13	3.64	3.86	1.58	1.95	3.42	2.36	1.70	2.29	1.92
ATT	1.20	0.40	0.58	0.86	1.50	1.94	0.20	1.30	1.14	0.23	0.30	0.88	1.08	0.18	0.70	1.22	1.50	1.94
ATC	0.60	2.60	2.31	1.82	1.25	0.79	2.20	1.30	1.14	2.31	2.40	1.59	1.62	2.65	1.93	0.94	1.23	0.88
ATA	1.20	0.00	0.12	0.32	0.25	0.27	0.60	0.40	0.72	0.46	0.30	0.53	0.31	0.18	0.38	0.84	0.27	0.18
GTT	0.42	0.17	0.12	0.46	0.52	0.80	0.00	0.72	0.82	0.18	0.32	0.36	0.80	0.22	0.62	1.33	0.98	1.29
GTC	0.84	0.35	1.58	1.23	1.16	0.93	0.80	0.92	0.71	0.73	0.96	1.58	1.44	1.00	0.98	1.09	0.80	0.39
GTA	0.63	0.00	0.24	0.62	0.39	0.13	0.10	0.31	0.47	0.00	0.16	0.36	0.00	0.22	0.00	0.12	0.09	0.39
GTG	2.11	3.48	2.06	1.69	1.94	2.13	3.10	2.05	2.00	3.09	2.56	1.70	1.76	2.56	2.40	1.46	2.13	1.94
TCT	0.67	0.00	0.90	0.96	1.39	1.50	0.26	1.16	1.50	1.83	1.62	1.36	1.20	0.40	1.32	1.61	1.46	1.50
TCC	1.00	1.33	1.95	1.92	0.92	0.94	2.09	0.97	0.94	1.30	1.62	1.47	0.69	2.13	1.61	0.29	1.64	1.33
TCA	1.33	1.33	0.45	0.24	1.15	1.13	0.52	1.36	1.13	0.52	0.46	1.36	1.20	0.40	0.73	1.76	0.73	1.33
TCG	0.67	0.67	0.15	0.24	0.23	0.00	1.30	0.00	0.19	0.26	0.23	0.45	0.17	0.27	0.15	0.29	0.36	0.00
AGT	1.33	0.00	0.60	0.72	0.46	0.38	0.00	1.16	1.13	0.78	0.69	0.45	1.37	1.20	0.59	1.46	0.91	1.00
AGC	1.00	2.67	1.95	1.92	1.85	2.06	1.83	1.36	1.13	1.30	1.39	0.91	1.37	1.60	1.61	0.59	0.91	0.83
CCT	2.00	0.29	1.14	0.77	1.33	1.16	0.53	1.37	1.18	0.71	0.44	0.86	1.50	0.67	0.93	1.39	0.80	1.04
CCC	1.60	1.71	1.71	1.81	1.73	1.68	2.11	1.03	1.18	2.35	1.56	0.86	1.50	2.10	1.20	1.57	1.47	1.39
CCA	0.40	1.14	0.69	1.29	0.67	0.90	0.84	1.60	1.65	0.00	0.44	1.43	1.00	0.86	1.20	1.04	1.07	1.39
CCG	0.00	0.86	0.46	0.13	0.27	0.26	0.53	0.00	0.00	0.94	1.56	0.86	0.00	0.38	0.67	0.00	0.67	0.17
ACT	1.26	0.00	0.69	0.92	1.33	1.33	0.64	0.59	1.00	0.43	0.50	1.19	0.94	0.80	0.61	1.39	0.97	1.04
ACC	1.26	2.22	1.79	2.00	1.33	1.33	2.40	1.33	1.38	2.29	2.50	1.19	1.88	2.27	1.74	0.87	1.52	1.19
ACA	0.84	1.33	1.10	0.77	1.19	1.00	0.48	1.78	1.38	0.71	0.83	1.33	0.71	0.53	0.70	1.57	1.10	1.33
ACG	0.63	0.44	0.41	0.31	0.15	0.33	0.48	0.30	0.25	0.57	0.17	0.30	0.47	0.40	0.96	0.17	0.41	0.44
GCT	0.86	0.76	0.39	0.89	2.59	1.78	0.88	2.00	1.52	0.91	1.05	1.38	1.52	1.13	1.04	0.97	1.24	1.04
GCC	1.14	1.71	2.97	2.07	0.71	1.33	2.15	1.40	1.33	1.37	1.47	1.79	1.14	1.54	1.57	1.52	1.71	1.04
GCA	1.71	0.76	0.52	0.89	0.71	0.89	0.49	0.60	0.95	0.34	0.42	0.83	1.14	0.82	0.78	1.24	0.67	1.63
GCG	0.29	0.76	0.13	0.15	0.00	0.00	0.49	0.00	0.19	1.37	1.05	0.00	0.19	0.51	0.61	0.28	0.38	0.30
TAT	0.00	0.25	0.20	0.38	1.27	1.17	0.67	1.38	1.41	0.43	0.25	0.71	0.93	0.29	0.65	1.24	0.74	1.05
TAC	2.00	1.75	1.80	1.63	0.73	0.83	1.33	0.63	0.59	1.57	1.75	1.29	1.07	1.71	1.36	0.76	1.26	0.95
CAT	0.80	0.25	0.77	0.56	1.00	0.83	0.75	1.00	0.67	1.00	0.89	1.00	0.40	0.29	0.57	1.20	1.00	1.20
CAC	1.20	1.75	1.23	1.44	1.00	1.17	1.25	1.00	1.33	1.00	1.11	1.00	1.60	1.71	1.43	0.80	1.00	0.80
CAA	1.00	0.18	0.08	0.44	0.63	0.71	0.36	0.67	0.75	0.00	0.10	0.67	1.00	0.11	0.56	1.07	0.46	0.63
CAG	1.00	1.82	1.92	1.56	1.38	1.29	1.64	1.33	1.25	2.00	1.90	1.33	1.00	1.89	1.44	0.93	1.54	1.37
AAT	1.11	0.00	0.56	0.53	0.77	0.77	0.50	1.00	1.11	0.40	0.00	0.93	1.20	0.60	0.91	1.48	1.13	1.18
AAC	0.89	2.00	1.44	1.47	1.23	1.23	1.50	1.00	0.89	1.60	2.00	1.07	0.80	1.40	1.09	0.52	0.87	0.82
AAA	1.06	0.14	0.39	1.17	1.20	1.14	0.17	1.11	1.15	0.33	0.20	0.74	0.75	0.29	0.86	1.05	0.83	1.26
AAG	0.94	1.86	1.62	0.83	0.80	0.86	1.83	0.90	0.85	1.67	1.80	1.26	1.25	1.71	1.14	0.96	1.17	0.74
GAT	0.46	0.24	0.35	0.40	1.00	0.86	0.44	1.23	1.11	0.42	0.36	1.10	1.40	0.38	0.89	1.39	0.80	0.80
GAC	1.54	1.77	1.65	1.60	1.00	1.14	1.57	0.77	0.89	1.58	1.64	0.90	0.60	1.63	1.11	0.61	1.20	1.20
GAA	1.00	0.35	0.15	0.73	1.03	1.13	0.20	1.23	1.10	0.00	0.07	1.22	0.57	0.48	0.89	1.19	0.92	1.32
GAG	1.00	1.65	1.85	1.27	0.97	0.88	1.80	0.77	0.90	2.00	1.93	0.78	1.43	1.52	1.11	0.82	1.08	0.68
TGT	0.86	0.75	0.86	1.60	0.92	0.92	0.67	1.43	0.80	0.40	0.33	0.93	0.93	0.55	0.44	1.56	1.46	0.92
TGC	1.14	1.25	1.14	0.40	1.08	1.08	1.33	0.57	1.20	1.60	1.67	1.07	1.07	1.46	1.56	0.44	0.55	1.08
CGT	0.00	0.00	0.00	0.38	1.11	1.14	0.62	0.27	0.52	0.00	0.20	0.00	0.55	0.63	0.32	0.00	0.24	0.64
CGC	0.57	2.40	1.64	1.50	0.67	0.29	4.00	0.27	0.52	1.86	1.80	0.92	1.09	1.58	1.26	0.71	1.20	0.21
CGA	1.14	0.00	0.18	0.94	0.00	0.00	0.31	0.55	0.52	0.41	0.40	0.46	0.00	0.79	0.63	2.12	0.72	0.86
CGG	1.14	1.80	2.36	1.50	1.11	1.14	0.62	0.55	0.52	1.66	1.20	1.85	1.09	1.58	1.90	0.00	1.20	1.29
AGA	1.43	0.00	0.36	0.75	2.44	2.86	0.00	2.73	2.61	1.03	1.40	0.46	1.09	0.79	0.00	2.12	1.20	1.71
AGG	1.71	1.80	1.46	0.94	0.67	0.57	0.46	1.64	1.30	1.03	1.00	2.31	2.18	0.63	1.90	1.06	1.44	1.29
GGT	1.11	0.38	0.41	0.52	0.14	0.29	0.61	0.31	0.44	0.27	0.30	0.67	0.75	0.71	0.64	1.19	0.67	0.86
GGC	1.56	2.29	1.79	1.29	1.29	1.29	1.33	0.92	0.74	1.47	1.63	0.89	1.25	1.96	0.96	0.59	1.20	1.14
GGA	0.44	0.19	0.55	1.16	2.00	1.86	0.49	2.15	2.07	0.67	0.74	1.11	1.25	0.62	0.96	1.78	1.20	1.43
GGG	0.89	1.14	1.24	1.03	0.57	0.57	1.58	0.62	0.74	1.60	1.33	1.33	0.75	0.71	1.44	0.44	0.93	0.57

**Table 3:** The t-test analysis between RSCU values of depression and housekeeping genes with 1000 bootstrap value, wherein iteratively resampling a dataset with replacement is involved.

S. no	Codon	t-value	p value (significance)
1	TTT	1.411	NS
2	TTC	1.411	NS
3	TTA	0.116	NS
4	TTG	0.787	NS
5	CTT	0.188	NS
6	CTC	1.204	NS
7	CTA	0.593	NS
8	CTG	0.04	NS
9	ATT	0.524	NS
10	ATC	0.75	NS
11	ATA	0.669	NS
12	GTT	1.138	NS
13	GTC	1.33	NS
14	GTA	3.58	***
15	GTG	1.8	0.064^#^
16	TCT	0.832	NS
17	TCC	0.122	NS
18	TCA	0.749	NS
19	TCG	0.599	NS
20	AGT	0.98	NS
21	AGC	0.04	NS
22	CCT	0.779	NS
23	CCC	1.77	0.08^#^
24	CCA	0.995	NS
25	CCG	0.636	NS
26	ACT	1.04	NS
27	ACC	1.59	NS
28	ACA	0.307	NS
29	ACG	0.964	NS
30	GCT	1.064	NS
31	GCC	0.113	NS
32	GCA	0.496	NS
33	GCG	0.488	NS
34	TAT	1.544	NS
35	TAC	1.544	NS
36	CAT	0.97	NS
37	CAC	1.22	NS
38	CAA	0.607	NS
39	CAG	0.607	NS
40	AAT	0.628	NS
41	AAC	0.887	NS
42	AAA	0.123	NS
43	AAG	0.123	NS
44	GAT	1.817	0.072^#^
45	GAC	1.817	0.0729^#^
46	GAA	0.671	NS
47	GAG	0.671	NS
48	TGT	0.416	NS
49	TGC	0.915	NS
50	CGT	1.53	NS
51	CGC	0.178	NS
52	CGA	1.352	NS
53	CGG	0.136	NS
54	AGA	0.468	NS
55	AGG	1.014	NS
56	GGT	1.121	NS
57	GGC	0.434	NS
58	GGA	1.213	NS
59	GGG	0.86	NS

*NS* non-significant.

***p < 0.0001.

^#^Significance level less than 10%.

### Relationship between codon bias, nucleotide skews and gene length

CUB had a significant positive association (r = 0.863, p < 0.001) with the length of proteins. We also investigated the relationship between protein length and protein expression level, but a lack of correlation was observed. Nucleotide disproportion is referred to as skews. Various skews, including AT skew, GC skew, purine skew, pyrimidine skew, keto skew, and amino skew are available to assess the effects of nucleotide disproportion on any parameter under consideration. Herein, we compared the effects of various skews on CUB, and found that only the pyrimidine, amino and keto skews had significant positive correlation with scaled Chi square value (SCS) values (r = 0.767, p < 0.05, r = 0.756, p < 0.01, r = 0.793, p < 0.01; Spearman correlation “r” with Bonferroni correction). Different nucleotide skew values are given in Table 4.

**Table 4:** Nucleotide skew in relation to the 18 depression associated genes.

Gene	AT skew	GC skew	Purine skew	Pyrimidine skew	Amino skew	Keto skew
*BDNF*	0.139	0.106	0.007	− 0.027	0.113	− 0.132
*COMT*	0.038	0.067	− 0.248	− 0.221	− 0.184	− 0.284
*CYP1A2*	0.000	− 0.075	− 0.144	− 0.217	− 0.217	− 0.144
*CYP2B6*	0.025	− 0.145	0.019	− 0.151	− 0.127	− 0.006
*CYP2C19*	0.020	− 0.034	0.111	0.057	0.077	0.091
*CYP2C9*	0.020	− 0.038	0.126	0.069	0.089	0.106
*CYP2D6*	− 0.082	− 0.061	− 0.290	− 0.271	− 0.345	− 0.214
*CYP3A4*	0.035	0.000	0.144	0.109	0.144	0.109
*CYP3A5*	0.058	− 0.024	0.160	0.079	0.136	0.102
*HLA-A*	0.111	0.051	− 0.250	− 0.305	− 0.201	− 0.350
*HLA-B*	0.115	0.043	− 0.251	− 0.318	− 0.210	− 0.356
*HTR2A*	− 0.027	− 0.078	0.107	0.056	0.029	0.134
*MC4R*	− 0.159	− 0.063	0.032	0.127	− 0.032	0.189
*MTHFR*	0.083	0.002	− 0.117	− 0.196	− 0.115	− 0.197
*SLC6A4*	− 0.086	− 0.020	− 0.091	− 0.025	− 0.111	− 0.005
*UGT2B15*	− 0.012	0.059	0.153	0.221	0.210	0.164
*UGT1A4*	− 0.042	0.005	− 0.038	0.009	− 0.033	0.004
*TPH2*	0.080	0.046	0.099	0.065	0.145	0.019

### CUB and gene expression profiling

Codon adaption index (CAI) is used as a quantitative method of predicting the level of expression of a gene based on its codon sequence^54^. In the study of Sahoo et al.^55^, critical analysis of predicted highly expressed (PHE) genes in *Arabidopsis thaliana* was performed by considering the expression data from Gene Expression Omnibus (GEO) datasets, where protein expression levels are quantified by RMA (Relative Molecular Abundance) signal intensity. The linear Pearson correlation coefficient between RMA and CAI showed a statistically significant correlation (r = 0.47, p < 0.05). In another experiment conducted by Guimaraes et al.^56^, protein abundance (PA) was measured for > 800 genes in. CAI was found to be significantly correlated with PA after controlling for mRNA abundance (r = 0.3526, P ≤ 0.001). The above examples clearly indicate that CAI might be conveniently used as a surrogate for protein expression. Thus, we used CAI values as expression data for depression genes (calculated through server CAIcal, developed by Puigbo and colleagues (2008) to correlate with their respective gene lengths^57^).

The CAI values of the genes associated with depression displayed values ranging from 0.713 (*UGT2B15*) to 0.85 (*CYP1A2*). The CAI value has a significant negative association with the SCS value (r = − 0.910, p < 0.001), and this indicates that in highly expressed genes, low codon bias is present^58^. A higher CAI indicated a relatively high protein expression level. Most of the AT ending codons have a significantly negative relationship with CAI, except for GTA, CGT, GCT (bearing no relationship with CAI). In contrast, most GC ending codons had a significant positive relationship with CAI, except for GTC, CTC, ACG, and TCG (with no relationship with CAI). The only exception was codon TTG that had a significant negative relationship with CAI.

### Codon context analysis revealed a context between stop codon UGA and other amino acid encoding codons

**Figure 3:**
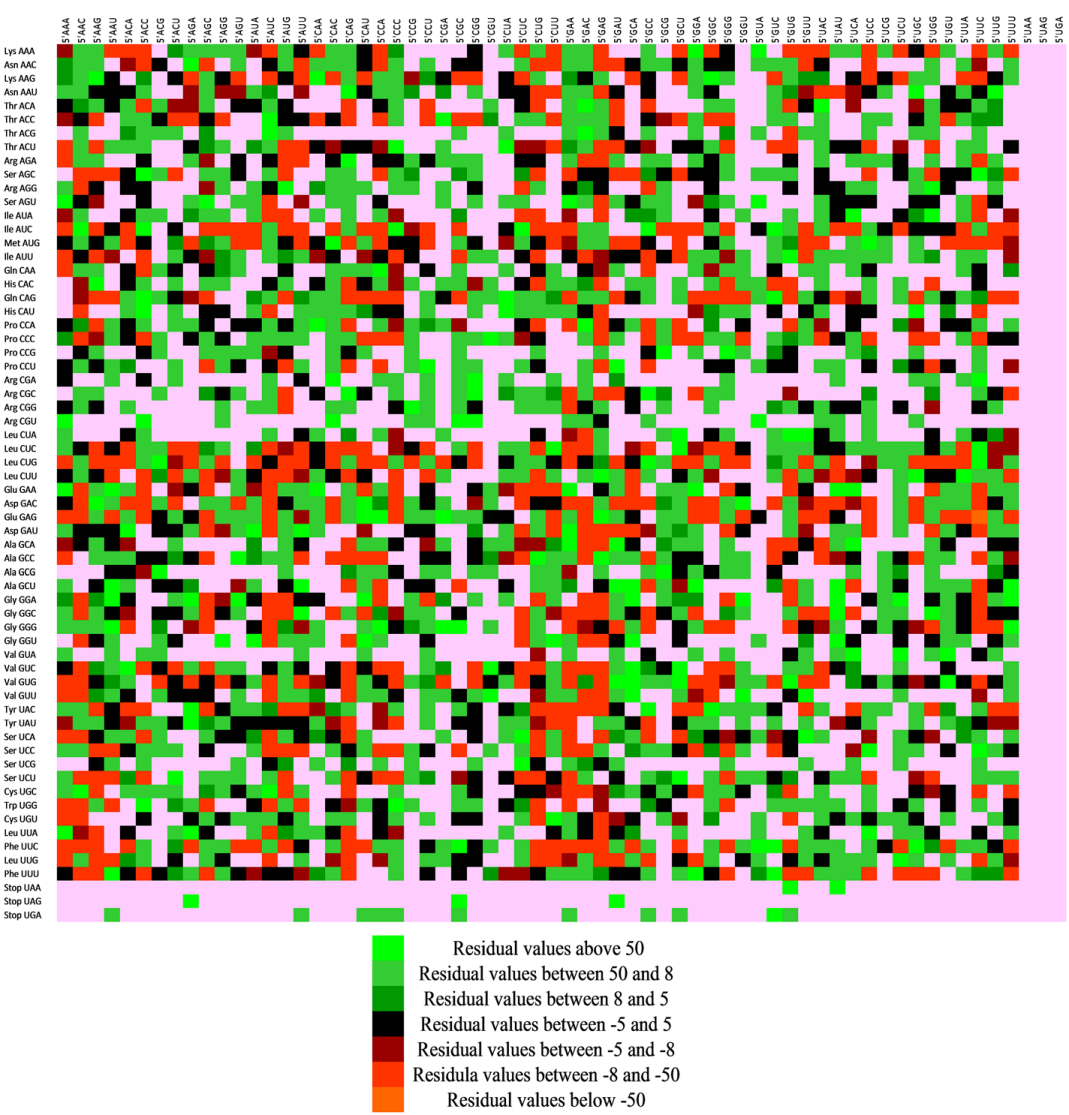
Codon context analysis for depression-associated genes. The green color portrays highly used codon pairs, whereas red represents lesser-used codon pairs. A pink color depicts a null usage of codons. Codon UGA and UAG were found paired with some specific codons. Statistically insignificant values are depicted as black.

On the one hand, where codon bias is a preferred use of codons, on the other hand, codon context refers to the presence of sequential pairs of codons in a gene^59^. In this light, codon context analysis was undertaken on the 18 genes associated with depression. Codon context, additionally, is a feature that influences the gene expression independent of codon bias^60^. The trend for codon context variation is depicted as a matrix of 64*64 codons. The total number of codon pairs observed in the 18 genes is 2047. As illustrated in Fig. 3, highly used codon pairs are displayed as a green colour, whereas lesser-used codon pairs are presented as red. The rows display 5’ codons, whereas the columns display 3’ codons (Fig. 3). It is clear from the Figure that stop codon UAG exhibited high context with many of the amino acid encoding codons. With that, all kinds of contexts (positive, negative and no context) were observed between the codons of envisaged genes.

### Arginine or proline initiated codon pairs are abundant

Out of 15 top overrepresented codon pairs, only two codons comprised either CpG or TpA as their part. Out of 540 rare codon pairs (absent codon pairs are excluded), a maximum of 75 codon pairs were arginine initiated, followed by 65 codon pairs for proline. Methionine-initiated codon pairs were rarest (09 only). Among the most preferred 15 codon pairs, a maximum of 04 were leucine initiated (Table 5). These results indicate a distinct pattern for codon pair preference or avoidance due to multiple evolutionary forces acting on depression-associated genes.

**Table 5:** Codon context analysis for top 15 overrepresented and rare codon pairs.

S. no	Overrepresented codon pair	Codon pair frequency	Rare codon pair	Codon pair frequency
1	GUG-GUG (VV)	28	GGC-CAA (GQ)	1
2	CUG-CUG (LL)	25	GGC-AUA (GI)	1
3	UUC-CUG (FL)	24	GGC-ACC (GT)	1
4	CUG-GCC (LA)	23	GGA-UUG (GL)	1
5	GAG-GAG (EE)	22	GGA-UGC (GC)	1
6	CUG-GAG (LE)	21	GGA-UCC (GS)	1
7	GUG-CUG (VL)	20	GGA-UAC (GY)	1
8	GCC-CUG (AL)	20	GGA-GUU (GV)	1
9	GCU-GUG (AV)	17	GGA-GUA (GV)	1
10	CUG-AAG (LK)	17	GGA-GAC (GD)	1
11	AAG-GAG (KE)	17	GGA-CGC (GR)	1
12	AAA-GAA (KE)	17	GGA-CAU (GH)	1
13	GUG-GAC (VD)	16	GGA-AGU (GS)	1
14	GCC-AUC (AI)	16	GCU-UGU (AC)	1
15	GAG-GCC (EA)	16	GCU-UGA (A-stop codon)	1

### Nucleotide disproportion influence on protein indices

We envisaged six nucleotide skews, namely AT skew, GC skew, purine skew, pyrimidine skew, keto skew, and amino skew. We performed Pearson linear correlation analysis between the nucleotide skews and protein properties to determine whether nucleotide disproportion influences physical protein properties (Table 6). Amino skew did not correlate with any of the protein properties envisaged. The results are suggestive of the effect of nucleotide disproportion on protein properties.

**Table 6:** Evaluation between nucleotide skew and protein properties.

	AT skew	GC skew	Purine skew	Pyrimidine skew	Amino skew	Keto skew	GRAVY	AROMA	PI	Instability index	Aliphatic index	Hydrophobicity	Acidic AA	Basic AA	Neutral AA
AT skew		*	NS	NS	NS	*	***	*	NS	NS	***	***	***	NS	NS
GC skew	0.552		0. NS	NS	NS	NS	NS	NS	NS	NS	NS	*	NS	NS	NS
Purine skew	− 0.074	− 0.152		***	***	***	NS	NS	*	NS	NS	NS	NS	NS	NS
Pyrimidine skew	− 0.319	− 0.019	0.917		***	***	NS	NS	*	NS	NS	NS	*	NS	NS
Amino skew	0.140	0.240	0.923	0.894		***	NS	NS	NS	NS	NS	NS	NS	NS	NS
Keto skew	− 0.487	− 0.365	0.907	0.938	0.750		*	*	*	NS	*	NS	**	NS	NS
GRAVY	− 0.897	− 0.444	0.198	0.428	0.021	0.553		NS	NS	NS	***	***	***	NS	NS
AROMA	− 0.478	− 0.336	0.346	0.418	0.208	0.507	0.381		NS	NS	NS	NS	NS	NS	NS
PI	− 0.167	− 0.256	0.575	0.520	0.468	0.571	0.163	0.080		NS	NS	NS	NS	NS	NS
Instability index	0.139	− 0.035	0.020	− 0.056	0.009	− 0.044	− 0.230	− 0.180	− 0.266		NS	NS	NS	NS	NS
Aliphatic index	− 0.780	− 0.439	0.177	0.356	0.003	0.484	0.932	0.162	0.143	− 0.249		***	**	NS	NS
Hydrophobicity	− 0.849	− 0.570	0.008	0.179	− 0.212	0.363	0.820	0.343	0.225	− 0.387	0.853		**	NS	NS
Acidic	0.826	0.361	− 0.285	− 0.511	− 0.140	− 0.601	− 0.868	− 0.443	− 0.373	0.296	− 0.679	− 0.607		NS	NS
Basic	0.062	0.037	0.270	0.240	0.281	0.211	0.024	0.024	0.151	0.029	0.059	0.126	0.050		NS
Neutral	0.005	0.213	0.109	0.185	0.188	0.101	0.106	0.169	− 0.184	0.195	− 0.149	− 0.419	− 0.353	− 0.233	

Lower triangle of matrix shows Pearson’s correlation coefficient, while the upper triangle shows the level of statistical significance. *p < 0.05, **p < 0.01, ***p < 0.001.

### Translation selection P2 is suggestive of a role of selectional forces

Translation selection (P2) values indicate the binding strength between the codon and anticodon. This was determined using the values of WWC, SSC, WWU, and SSU using the average RSCU values, and a value of 1.01 indicates strong selectional forces behind it.

### Neutrality analysis confirms major role of selectional forces

**Figure 4:**
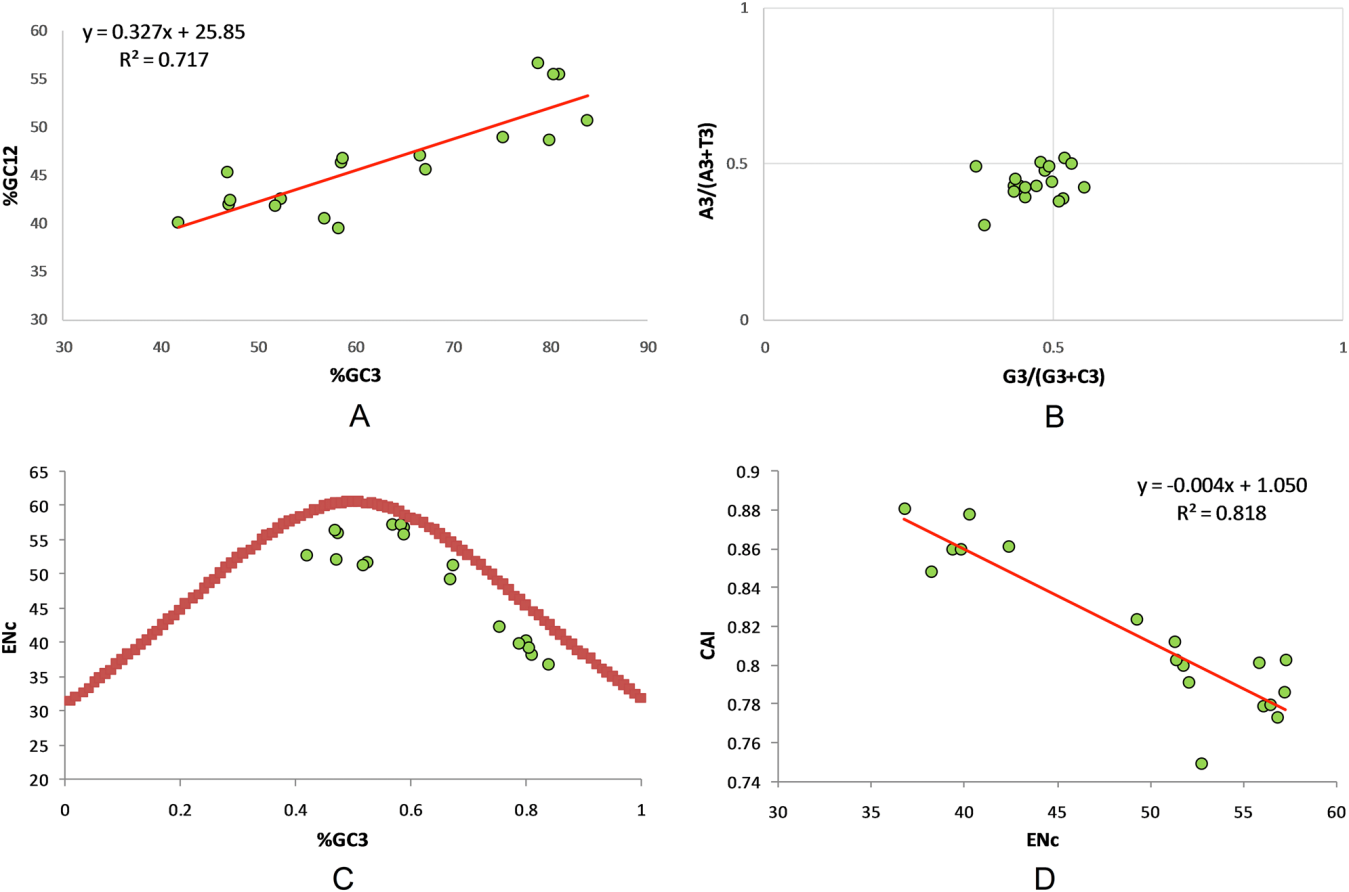
(**A**) Regression analysis between average %GC content at codon position one and two (%GC12) and %GC (%GC3) content at the third codon position. (**B**) Parity plot comprising GC bias (G3/G3 + C3) on abscissa and AT bias (A3/A3 + T3) at the ordinate. (**C**) ENc-GC3 analysis showing presence of data points below the expected Nc curve depicting prevalence of selection force. (**D**) Regression between CAI and ENc (effective number of codons) revealed that 81.81% variations in CAI are attributed to ENc and thus on codon bias.

Regression analysis between the %GC3 and %GC12 provided a slope value of 0.3276, which indicated that relative neutrality was 32.76% and the relative constraint was 67.24% (Fig. 4A). This signifies that selectional force (67.24%) was dominant over mutational force (32.76%). The graph also indicates that %GC3 is responsible for 71.7% variation in %GC12. Additionally, %GC12 and %GC3 are significantly positively correlated (r = 846, p < 0.001).

### Parity analysis revealed preference of T and C over A and G nucleotides

Parity analysis determines the bias between A/T and C/G at the third codon position. At the center, where the axis value is zero, A = T and C = G. In the present study, the average position of x = 0.469 ± 0.050 (AT bias) and y = 0.439 ± 0.054 (GC bias). A bias value of less than 0.5 indicates a preference for pyrimidine over purines^61^. Herein, our analysis indicated that thymidine is preferred over adenine, and that cytosine is preferred over guanosine (Fig. 4B).

### Relationship of codon bias with %GC3 content and gene expression

An ENc (effective number of codons) versus GC3 plot is generally used to study the effect of %GC3 composition, which is suggestive of both a mutational force and compositional parameter on codon bias. In the event that codon choice is constrained by mutational force alone, all the data points will lie on or just below the GC3 curve, whereas in the case of an operating selection force, the data points are well below the GC3 curve^62^. In the present study, only a few points were present near the curve. The rest of the data points are present below, suggesting selection force as a dominant force in shaping codon usage in depression-associated genes (Fig. 4C). Furthermore, we investigated the effect of codon bias on gene expression by regressing them. Since ENc is the non-directional measure of codon bias, a negative correlation between them (Pearson correlation r = − 0.904, p < 0.0001) indicates that gene expression also increases with increasing codon bias. Overall, 81.81% variation in gene expression is attributed to codon bias (Fig. 4D).

### Effects of mutation pressure on codon composition is highest for G and least for T nucleotide

**Figure 5:**
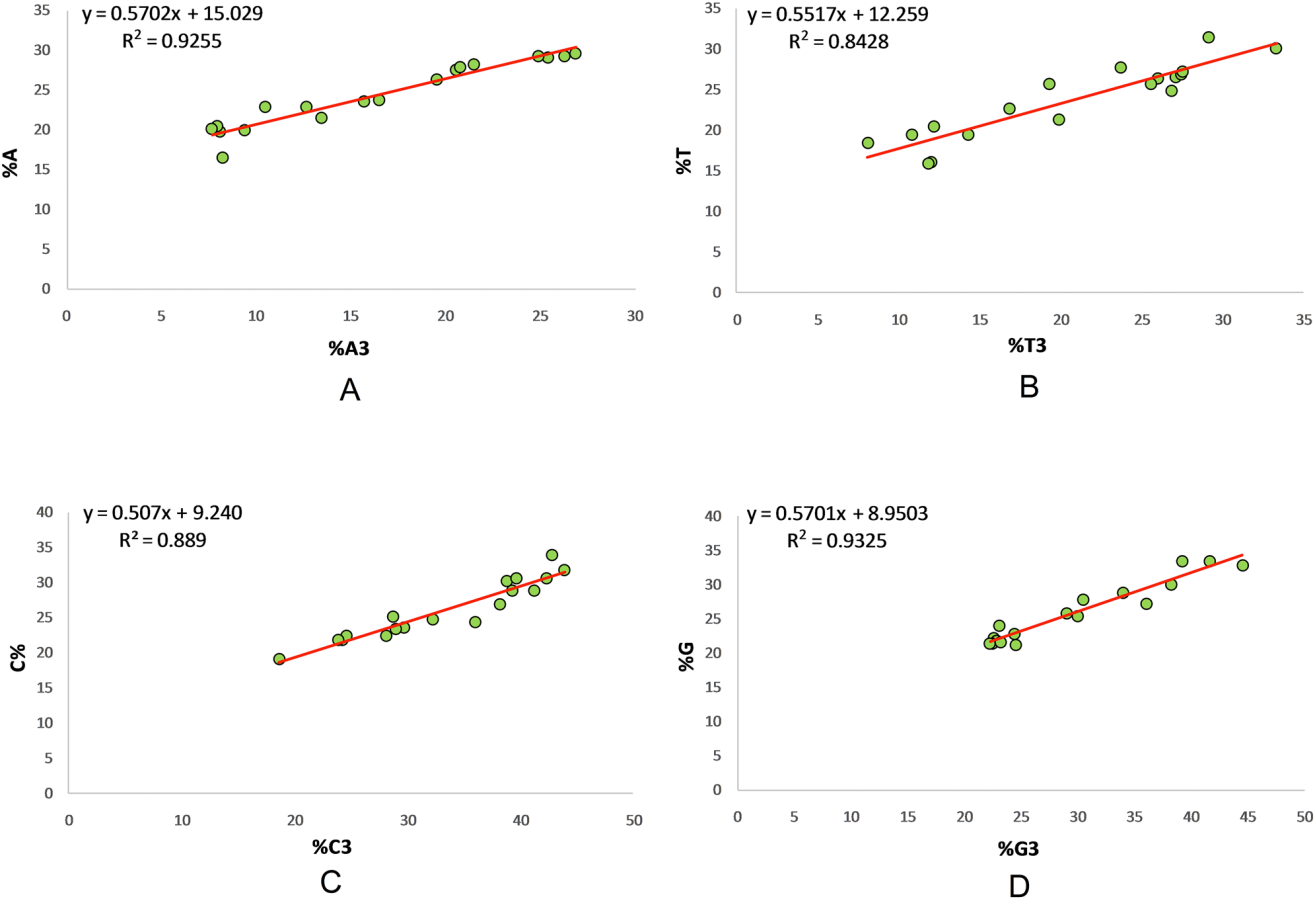
Regression analysis between overall nucleotide content and content at the third codon position. Panel (**A**): A3 and A; panel (**B**): T3 and T; panel (**C**): C3 and C; panel (**D**): G3 and G.

Mutation at the third position of a codon did not change the meaning of the codon, with regard to the amino acid encoded by it, and is called the silent position of the codon because of redundancy of the code. Nevertheless, this position is affected by mutation force since, here, mutation changes the nucleotide but not the meaning of the codon. The effect of mutational force on composition was 92.55%, 84.28%, 88.9%, and 93.25% for nucleotides A, T, C, and G, respectively (Fig. 5). In this regard, it is clear from Fig. 5 that mutational forces on G nucleotide contributed the most in relation to determining its composition (93.25%), whereas mutational forces on nucleotide T contributed least towards determining its composition (84.28%).

### Principal component analysis

**Figure 6:**
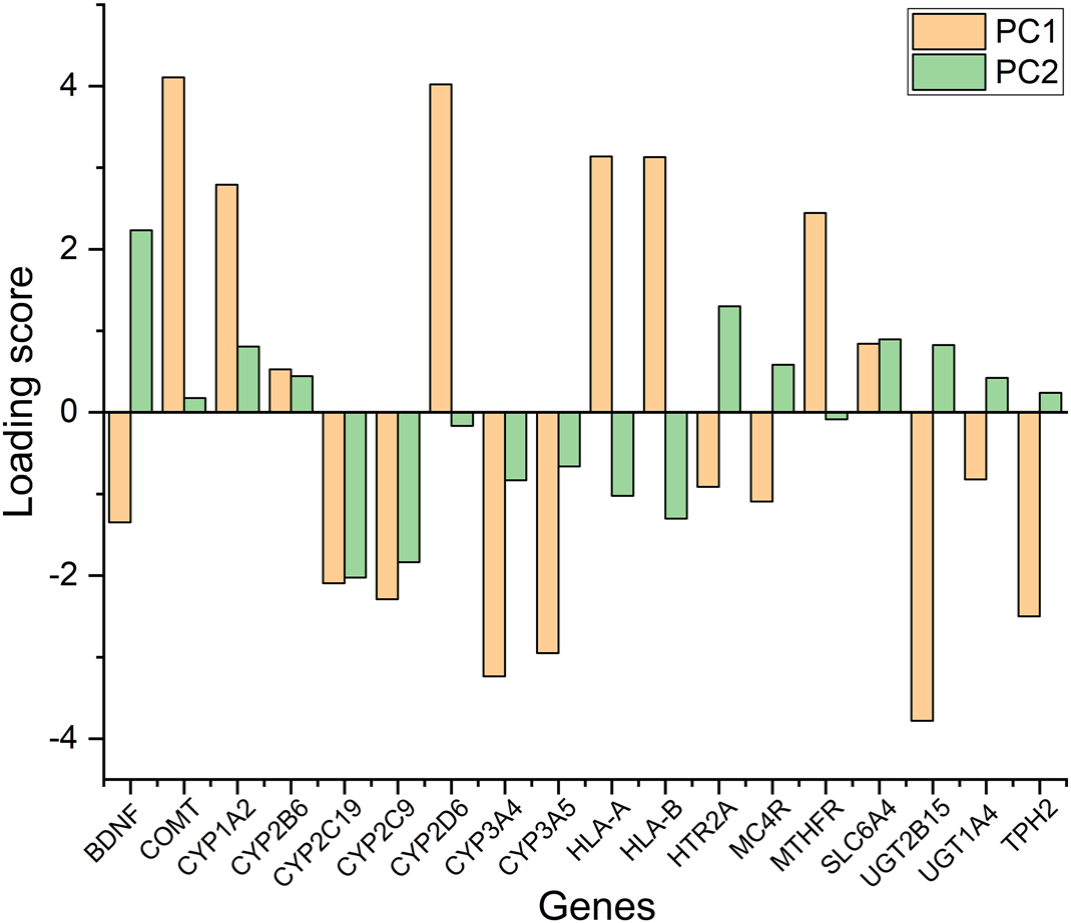
PCA analysis for 18 depression associated genes. Orange and green bars depict the loading scores of PC1 and PC2 for the genes.

Principal component analysis was undertaken using the 59 RSCU values of 59 codons. Figure 6 represents the correspondence analysis and reveals that the first two axes account for significant variation (50.46% and 10.88%, respectively). The third and fourth axes account for 6.64% and 5.78% variation, respectively, and the contribution of the first four axes is 73.76%. Based on the loading values, codons AGA, CTG, CGC, and GGA influence CUB the most in depression-associated genes. The first and second principal component (PC1 and PC2) scores of different genes are provided in Fig. 6.

## Discussion

Depression is a disorder with a wide range of symptoms. In evaluating patients with depression, GWAS has revealed a high degree of polygenicity that underlies the mental illness and related complex phenotypes, and has discovered that many SNPs with relatively small effect size, when combined, potentially contribute to phenotype development^4^. Polygenicity includes some genetic heterogeneity; affected people may have different combinations of risk alleles, and unaffected people will also carry many of these variants. Depression is clearly a heterogeneous condition, as evidenced by the fact that two people can be diagnosed with depression but have no common symptoms. Added to this, neurodegenerative disorders too^63^ can potentially contribute to depression^64^.

In this light, various studies have been undertaken to understand the physiology and genetics behind depression. To our knowledge, however, no previous work has described the compositional features and codon usage patterns of genes associated with depression. Hence, the present research focuses on the codon usage of genes associated with depression. Our evaluation used a panel of 18 genes that have been associated with depression (Table 1). Although this number is not optimal and can be considered by some to be undersized for statistical analyses, it is the maximum number of genes available for depression detection from the NCBI gene testing registry. The products of genes are involved in multiple biological functions and pathways (given in Table 1), and altered expression levels or SNPs can lead to various genotypes that result in diseased conditions or different response to medications.

Nucleotide composition is imperative in knowing the codon usage since many of the parameters associated with codon usage indices, including nucleotide skew, neutrality, and parity plots, are composition dependent. Compositional analysis revealed that %C occurrence was highest, with the lowest occurrence of %T. The %GC3 content was the most variable compositional parameter and varied between 41.80 and 83.82%.

CAI is a measure of gene expression level, and this measure compares the codon composition of a gene with a reference set of genes^65^. Our study found a range of CAI values between 0.713 (*UGT2B15*) and 0.85 (*CYP1A2*). In *Escherichia coli* (*E. coli*), which has long been regarded as a model organism in the study of CUB, the highest CAI value of 0.85 was for the lpp gene, one of the most abundant genes, encoding an outer membrane lipoprotein^66^. Hence, it can be speculated that the CAI value 0.85 (CYP1A2 gene), in our depression study, likely also is associated with a high-level expression. The relationship between the CAI and expression value can be better understood in the light of an experiment conducted by Dos Reis et al.^58^, who distributed the *E. coli* genes into three groups based on codon usage and expression level data obtained from microarray experiments. They found a positive relationship between the CAI value and expression level in one of these group. In another group, the genes with low CAI were highly expressed, which contradicts the set paradigm of CUB, where optimal codon usage leads to higher CAI. However, the results are still explainable based on the mutation-selection balance hypothesis of codon usage. High CAI values were also obtained in the present study, indicating a higher expression level. However, other dynamic factors, including mutational-selectional balance, could provide attributing factors to the expression. CAI is associated with compositional constraints and can potentially show all relationships (negative, positive, and no correlation). Hence it can be inferred from this study that the gene expression level depends on the base composition. Such a phenomenon could be the compositional pressure on CUB, which ultimately drives the gene expression. Our view is supported by the results of Sahoo et al.,^67^ who described the critical role of codon composition in regulating the gene expression profile in the *Arabidopsis thaliana* genome (a small plant from the mustard family native to Eurasia and Africa) based on the score of modified relative codon bias. A study by Franzo and colleagues^68^, likewise, demonstrated that CUB is highly affected by nucleotide composition in an evaluation of an infectious bronchitis virus. The genes associated with depression showed an interesting pattern related to nucleotide composition and CUB. After comparing compositional constraint relationships with SCS, one of the measures of CUB, we found a negative relationship of SCS with G nucleotides (overall %G, %G2, %G3 and %GC2) only. This signifies the importance of G nucleotide in determining codon usage.

Codon usage bias is affected by several factors, and gene length is one of them. Based on a study on codon usage in 8,133, 1,550, and 2,917 genes, respectively, from *Caenorhabditis elegans*, *Drosophila melanogaster* and *Arabidopsis thaliana*, a significant negative linkage between codon usage and protein length was explained^69^. On the other hand, Eyre-Walker^70^ found a positive association between codon usage and gene length, suggesting selection against missense errors in *E. coli*. In this light, it can be inferred that length can have both positive and negative impacts on CUB—depending on the model organism under evaluation. CUB and protein length were positively correlated with GC3 content and the correlation was stronger for %GC12 content in all the proteins envisaged, without any exception. Our results agree with Khandia et al.^71^, who found that in all the proteins whose size ranged between 150 and 3000 amino acids in a study focused on primary immunodeficiency and cancer, GC12 content was lower than GC3—without any exception.

In our current study, dinucleotides CpG, GpT, and TpA were underrepresented, whereas ApG, CpT, GpA, and TpG were overrepresented. In the human ORFome (open reading frames within a genome), CpG and TpA dinucleotides show the highest level of suppression, and GpT is the third of those with the lowest abundance^72^. Thus, it appears that depression-related gene sets also follow the common trend of odds ratio present in human ORFome. CpG dinucleotides occur at a low frequency in the human genome, and this is attributed to a higher mutation rate of 5-methylated CpG to TpG, and, as a result, the TpG dinucleotide is increased^73^. Contrary to the results of Kunec and Osterrieder^72^ and to ours, Franzo et al.^68^ found an overrepresentation of GpT dinucleotide. ApG, CpT, GpA, and TpG overrepresentation partially concord with Franzo et al.^68^, who reported ApG and TpG dinucleotide pairs overrepresented in the whole-genome, and CpT in the polyprotein region only in infectious bronchitis virus. Such results suggest that the odds ratio might serve as a molecular signature^74^.

RSCU analysis indicated that GC ending codons were preferred over AT ending codons; however, parity analysis indicated that T and C nucleotides are preferred over A and G nucleotides. In accordance with the results of nucleotide analysis, codons encompassing TpA and CpG dinucleotides (TTA, GTA, ATA, CTA, CGT, ACG, GCG, CCG, TCG) were underrepresented. The overrepresentation of CTG and GTG codons observed in the present study matches the results of Khandia et al.,^71^, who found overrepresentation of CTG and GTG in 78.33% and 68.33% of genes common to primary immunodeficiency and cancer, respectively. This abundance of CTG and GTG codons might have come from the conversion of CpG to TpG dinucleotide, an integral part of the CTG and GTG codons. Such result suggest that RSCU bias is the result of dinucleotide bias^72^, resulting from a consequence of intrinsic characteristics and evolutionary forces like selection and mutation^75^.

The codons also influence the gene expression level, and it was observed that most AT-ending codons have a negative association with CAI. In contrast, most GC ending codons have a positive association with GC ending codons. The only exception to this was the codon TTG, which is negatively associated with CAI. The two codons, AGG and TTG, behave differently in the human genome. When the other C and G ending codons are decreased, these two increase^76^, which is probably why they are inversely affected by CAI.

Compositional properties affect codon usage and nucleotide disproportion too. Nucleotide disproportion (skews) also affects CUB and, in the Nipah virus, an association between CUB and nucleotide skew similarly has been reported^77^. We found CUB becomes affected by purine skew. Various skews significantly affected different protein indices, also suggestive of the role of compositional constraints on the physical properties of proteins. In mitochondrial NADH dehydrogenase genes (ND genes, encoding for respiratory complexes) of Amphibia, amino skew, purine skew, and keto skew showed a significant correlation with ENc, thereby demonstrating that skewness can potentially affect the CUB^78^. In the genes associated with depression, %GC12 and %GC3 are found to be significantly positively correlated (r = 0.846, p < 0.001), and this correlation is suggestive of the role of mutational force in shaping codon usage^79^.

The CUB and codon context bias are important parameters to be considered during heterologous protein expression^80^. In our study, it was evident that few of the codons remain minimally used, and this is in accord with the studies of Chakraborty et al.,^81^ on codon context in leukemia-associated genes. Identical codon pairs, GTG-GTG and CTG-CTG codon pairs were the most favored codon pairs in the depression-associated gene set. Here, Co-tRNA and identical codon pairing help conserve the resources and enhance translational efficacy by up to 30%^82^.

In the present study, we performed gene correlation analysis to determine whether the genes involved in similar functions share similar attributes or not. Gene correlation analysis was undertaken based on RSCU to determine whether genes have a similar kind of codon usage or not. The data indicated that all the 18 genes evaluated displayed similar codon choices, as evidenced by the positive relationship among all the genes in the study. However, the correlation value varied at different levels, and few genes did not display correlation. When the gene correlation was studied at the protein indices level, all genes were found positively correlated except for the *CYP3A4* gene, which showed no correlation with any of the genes. Such analysis helps determine how genes involved in one kind of ailment may be similar based on different parameters, and we found similarities between them based on RSCU and protein indices.

Translational selection (P2) refers to the strength of the binding force between the codon and anticodon, and indicates selectional pressure. In the four cotton species (*G. arboreum*, *G. raimondii*, *G. hirsutum* and *G. barbadense*), P2 values were more than 0.5. In this light, our result indicates the dominant role of selection over mutation pressure in the codons’ usage^83^.

Upon evaluating the effects of mutational forces on overall nucleotide composition, it was evident that mutational pressure affected nucleotide A and G equally (approx. 57%), whereas nucleotide C was least affected. Principal component analysis indicated that the codon usage by genes is majorly influenced by G and C ending codons. Overall analysis revealed the importance of compositional, mutational, and selectional pressure. However, the role of selection pressure was dominant over the others^84^. There are a few striking similarities in neurobiological alterations between depressive disorders and neurodegeneration, as in Alzheimer’s, Parkinson’s, and Huntington’s disease^64^. In the study of Khandia et al.,^63^, codon pattern studies in neurodegeneration-related gene sets have been undertaken with minor overlap in which gene composition, dinucleotide analysis, RSCU, CAI, and different protein indices were evaluated. In the future, parameters like codon pair occurrence, codon context, and effects on gene expression on codon bias might be investigated in such genes.

The present study envisages an investigation of different molecular patterns and relative synonymous codon usage in 18 depression-associated genes; here, out of 18 genes, 09 genes showed modulation of gene expression during the depressive state. *BDNF*, *COMT*, *CYP2C9*, *CYP3A4*, *HTR2A*, *SLC6A4*, and _MTHFR_ genes showed reduced expression, while *UGT1A1* and *CYP2C19* showed enhanced expression. For other genes, different genotypes (related to SNPs) associated with depression or response to depression therapy could not be included in the study since the SNPs responsible for depression might be present in promoter/repeats/exon/ intron/leader sequences^85^, but the analysis of codon usage, codon pair, CAI, and other patterns is intended for only protein-encoding sequences. As a consequence, we acquired only the coding sequences of the envisaged genes, which were available as RefSeqGene in the NCBI database. In relation to the 07 genes whose expression is found downregulated during depression, this theoretically might be corrected for their expression level by introducing a copy of the gene (such as by using gene therapy methods employed currently, like CRISPR-cas) with codon usage in such a manner so that codons with lower RSCU values might be changed with codons having higher RSCU values, to enhance the gene expression which might be presumed using the index CAI; thereby using the current study to open potential new hypotheses and avenues for future research.

## Conclusion

In relation to CUB evaluation of depression associated genes, compositional analysis revealed that %C nucleotide was highest, followed by %G, %A, and %T. Among all compositional constraints, %GC3 was variable the most. All the 18 genes envisaged in the study had high CAI values, indicating high-level gene expression. Additionally, within the present study, the gene expression level was driven by compositional constraints. Interestingly, CUB in depression-linked genes is associated solely with overall G nucleotide composition and composition at the second and third codon position, referring to the effect of G nucleotide compositional constraint on CUB. Codon bias was positively correlated with the length of the gene, indicating increased bias with the length of the protein. CpG, GpT, and TpA dinucleotides were underrepresented with an over-representation of ApG, CpT, GpA, and TpG dinucleotides. The pattern present in dinucleotides was seen further in RSCU values of codons, where all CpG and TpA containing codons have low RSCU values and are underrepresented. Likewise, overrepresented dinucleotide CpG is further exhibited in CTG and GTG over presented codons. Among the nucleotide skews evaluated, purine skew was found to affect CUB. A highly significant positive relationship between GC3 and GC12 indicated the role of mutational force in shaping codon usage. The neutrality plot exhibited the prominent role of the selection force in shaping codon utilization. The parity plot results further supported this notion in which T and C nucleotides are preferred over A and G nucleotides. Based on translation selection (P2) analysis, it could be inferred that the genes had low codon bias. Gene correlation analysis based on RSCU revealed a variable degree of positive correlation among genes showing a similar codon usage pattern, which the PCA further established. All the genes clustered together indicated a similar codon choice. Codon context analysis revealed the abundance of identical codon pairs GTG-GTG and CTG-CTG, which enhance the translational rates and are results of selection forces. Based on the study, a synthetic construct could potentially be synthesized with the information on relative synonymous codons, codon bias, codon pair bias, and CAI in hand. Such a construct might help modulate gene expression. For example, in 07 genes studied here, which are downregulated during depression, restoring an overexpressing copy within the body through gene therapy might potentially curb the ailment, and provides an hypothesis and potential avenue for future research.

## Material and methods

### Pathway analysis

For the envisaged genes, pathway analysis was conducted through PANTHER knowledgebase. The database provides comprehensive information regarding the evolution of protein-coding gene families. The database was retrieved from the weblink https://www.pantherdb.org/.

### Compositional analysis (overall and at various positions of codon)

A panel of a total of 18 gene sequences targeted to a depression diagnosis was available from the Genetic Testing Registry, National Center for Biotechnology Information Search database (gtr/tests/508,961 by Assurex Health Inc, gtr/tests/569,407 by Genomind Professional PGx Express CORE Anxiety & Depression, and gtr/tests/579,485 by Intergen Genetic Diagnosis and Research Centre). Each of the genes could have had different isoforms /genotypes; hence we acquired the 'reference' gene sequences (RefSeqGene) from the National Center for Biotechnology Information Search database, and the feature 'CDS' was selected, converted into 'fasta format' and used for further studies. Information regarding these genes is given in Table 1.

The composition of nucleotides affects various codon usage parameters. The overall nucleotide composition of individual nucleotides and their composition at all of the three positions of codons for these 18 genes were determined using the software CAIcal developed by Ref.^57^. The average percent of GC composition at the first position (%GC1) and the second position (%GC2) viz. %GC12 and GC3 were used in neutrality analysis. %AT and %GC compositions at third codon positions were used in parity analysis.

### Dinucleotide odds ratio analysis

The odds ratio is the ratio between the observed and expected frequency. An odds ratio below 0.73 is indicative of under-representation, whereas values above 1.23 indicate over-representation of any dinucleotide pair^62^.

### Relative synonymous codon usage (RSCU) analysis

The RSCU is the ratio of the observed frequency of synonymous codons and is calculated using the formula

$$RSCU = \frac{Xij}{{1/ni\sum\nolimits_{j = 1}^{ni} {Xij} }}$$

where *Xij* stands for the frequency of the *j*th codon for *i*th amino acid and *ni* is the number of codons for the *i*th amino acid (*i*th codon family).

RSCU values of less than 0.6 are considered underrepresented codons and RSCU values above 1.6 are deemed over represented codons^86^.

### Determination of scaled Chi square value (SCS)

The SCS, unlike the effective number of codons (ENc), is a directional measure of CUB^87^. SCS values were calculated for each of the genes implicated in depression.

### Codon adaptation index (CAI)

CAI is a measure of CUB and helps determine the gene expression level. The CAI value ranges between 0 and 1, and the higher the value, the higher the expression^65^. CAI values are adjusted in the synthetic biology approach to obtain maximum expression level.

### Skew calculation

Skew, herein, is a disproportionate use of nucleotides. Asymmetrically biased nucleotides arise due to asymmetric replication with leading and lagging strands^88^. AT skew, GC skew, purine skew, pyrimidine skew, keto skew, and amino skews were determined.

### Estimation of physical properties of protein

pI or isoelectric point, instability index, aliphatic index, hydrophobicity, frequency of acidic, basic, and neutral amino acids, GRAVY, and AROMA, are the physicochemical properties of a protein that were assessed in the present study to evaluate the effects of various parameters on protein properties. Theoretical pI (PI), instability index (II), aliphatic index (AI) and hydrophobicity (HY) were computed using the ProtParam tool—ExPASy^89^. The frequency of acidic, basic, and neutral amino acids was determined using the Peptide2 tool available at Peptide 2.0 Inc.

### Regression analysis

A regression analysis between %GC3 and %GC12 defines the magnitude of mutational and selection forces. If the slope tends to be near 1, it indicates that mutational force solely influences the codon usage and vice versa^90^. Simultaneously, a perfect correlation between GC12 and GC3, with a slope near value 1, indicates mutational force as the dominant one^91^.

### Parity analysis

A parity rule 2 (PR2) bias indicates the bias between A and T and C and G at the third position of the codon. A parity plot is made by plotting AT bias [A3/(A3 + T3)] as the ordinate and GC bias [G3/(G3 + C3)] as the abscissa^79,92^.

### Translational selection

The P2 analysis indicates the strength of codon-anticodon interaction and indicates translation efficacy when information of a preferred codon set is unknown^83^.

Translational selection P2 was calculated using the formula:

$${\text{P}}2 = ({\text{WWC}} + {\text{SSU}})/(WWY + SSY)$$

where W = A or U, S = C or G, and Y = C or U.

Moreover, any values above 0.5 indicate a bias favoring translational selection^93^.

### Codon context analysis

In prokaryotic genes, it was first observed that codons and codon pairs also exhibit a bias in occurrence^94^. In another study, it was observed that codon pairs also influence the rate of translation. Overrepresented codon pairs are translated at a slower speed than pairs of underrepresented codons. The phenomenon is related to the compatibilities of adjacent tRNAisoacceptor molecules present on ribosomes participating in translation. Such results suggest co-evolution of frequency of one codon to the next codon with structural compatibilities and tRNAisoacceptor abundance as a measure to control translation rates^95^. Furthermore, codon pair optimization and deoptimization have been proven to affect the translation efficiency in several experiments deciphering the importance of codon context bias^96,97^. We performed codon context analysis using Anaconda 2 software in the present study.

### Statistical analysis

Statistical analyses, such as Pearson correlation and regression analysis, were undertaken using PAST4 software. Standard calculations, such as additions and subtractions, were performed in Microsoft Office 2010 used in skew and other analyses. Principal component analysis was undertaken using PAST4 software.

### Supplementary Information

Supplementary Information.

## Data Availability

Available upon request from RK.
